# Mesoporous Semi‐Permeable Flexible Polyurethane Membranes: Advancing Bioartificial Pancreas Design for Type 1 Diabetes Treatment

**DOI:** 10.1002/marc.202500049

**Published:** 2025-02-14

**Authors:** Bryan Gross, Emeline Lobry, Séverine Sigrist, Elisa Maillard, Jordan Magisson, Charles‐Thibault Burcez, Manuel Pires, Anne Hébraud, Guy Schlatter

**Affiliations:** ^1^ Institut de Chimie et Procédés pour l'Energie, l'Environnement et la Santé, ICPEES UMR 7515, CNRS Université de Strasbourg, Ecole Européenne de Chimie, Polymères et Matériaux 25 rue Becquerel Strasbourg Cedex 267087 France; ^2^ Defymed, 9 rue Albert Calmette Strasbourg 67200 France; ^3^ Institut National de la Santé et de la Recherche Médicale, UMR_S 1121 Université de Strasbourg, Faculté de Chirurgie Dentaire Strasbourg France

**Keywords:** diabetes, green electrospinning, mesoporous membrane, nanofibers, waterborne polyurethane

## Abstract

This study reports the development of elastomeric mesoporous polyurethane (PU) membranes for bioartificial pancreas applications in type 1 diabetes treatment. The membranes are designed to exhibit semi‐permeable properties, enabling insulin diffusion while restricting larger immune molecules, such as immunoglobulin G (IgG). Although electrospinning is a widely used technique for fabricating porous membranes for controlled drug release, it typically results in an average pore size on the order of few micrometers, which is two orders of magnitude larger than the mesoporous scale required. In this work, a green‐electrospinning process using waterborne PU suspension and poly(ethylene oxide) (PEO) is employed, followed by thermal annealing and washing steps. The resulting membranes exhibit a controlled pore size in the mesoporous range (≈20 nm measured by capillary flow porometry). Diffusion tests confirmed selective permeability, with a recovery rate of 25% for insulin and a recovery rate below 5% for IgG, meeting therapeutic needs. In vivo characterizations show no degradation and good biocompatibility of the membranes without chronic inflammation. Moreover, mechanical characterization demonstrates the membranes' flexibility and strength, making them suitable for minimally invasive surgical implantation. These findings underscore the potential of PU membranes for long‐term biomedical applications, addressing critical challenges in permeability and mechanical stability.

## Graphical abstract

Elastomeric mesoporous membranes are developed for the elaboration of a bioartificial pancreas for type 1 diabetes treatment. Using green‐electrospinning followed by thermal and washing post‐treatments, the membranes achieve ≈20 nm pore sizes, biocompatibility, and mechanical strength and flexibility. Selective permeability enables insulin diffusion (25%) while retaining IgG antibodies (<5%). These features highlight their potential for implantation by minimally invasive surgery.

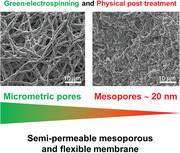

## 1 Introduction

Type 1 diabetes, as defined by the World Health Organization, is considered as a chronic metabolic disorder that affects the regulation of glycemia. In a healthy individual, the pancreas produces an adequate level of insulin, an essential hormone that allows cells to absorb glucose present in the blood and, thus maintaining glucose at a balanced level in blood. However, in the case of patients suffering from type 1 diabetes, the immune system attacks the pancreatic beta cells being responsible for insulin production, which results in insulin deficiency and inadequate blood sugar self‐regulation.^[^ ^1^ ^]^ Chronic hyperglycemia can ultimately lead to serious complications, such as the risk of stroke, blindness, or even kidney failure. A promising approach for the treatment of type 1 diabetes relies upon the implantation, such as in the extra‐peritoneal space, of a macroencapsulation device^[^ ^2^, ^3^ ^]^ embedding beta cells in order to produce and deliver insulin in a controlled manner.^[^ ^4^ ^]^ This macroencapsulation device, the so‐called bioartificial pancreas, allows to isolate the cells from the recipient's immune system. Without the risk of rejection, it is then conceivable to use cells derived from various sources, such as stem cell‐derived cells.^[^ ^5^ ^]^ Genetically modified cells or even cells of animal origin (i.e., xenotransplantation) are also being considered for this purpose.^[^ ^6^, ^7^ ^]^ A bioartificial pancreas acts as a pouch that isolates transplanted insulin‐secreting β cells from the recipient's immune system. Additionally, it should protect the recipient from foreign cells while enabling efficient and rapid exchanges between these cells and the bloodstream to achieve optimal glycemia regulation. To prevent rejection or inflammation, the device must fulfill four key requirements: biocompatibility, non‐biodegradability, immunoprotection, and semi‐permeability.

The semi‐permeability enables the diffusion of essential molecules as oxygen and glucose (i.e., cell's nutrients) and insulin while restricting the diffusion of larger immune system compounds such as IgG antibodies. As the molecular diameter of insulin is of 2 nm^[^ ^8^ ^]^ and the one of IgG is of 11 nm,^[^ ^9^ ^]^ the selective diffusion of these two compounds can be envisaged considering their steric hindrance.^[^ ^10^ ^]^ Indeed, Magisson et al.^[^ ^10^ ^]^ developed a macroencapsulation device made from a track‐etched polyethylene terephthalate (PET) membrane with a mean pore size of 11–16 nm. They demonstrated effective semi‐permeability by allowing the diffusion of insulin with a recovery rate of 34.3% while restricting the diffusion of IgG antibodies (recovery rate of 10.4%). Nonetheless, such PET device is rigid, and the implantation requires traditional open surgery.

The present study proposed a novel approach for manufacturing a mesoporous membrane (i.e., average pore size ranging from 2 to 50 nm) that can be easily assembled through welding to form the device that encapsulates the insulin‐secreting β cells with a design similar to what it was proposed by Magisson et al.^[^ ^10^ ^]^ The membrane is designed to be sufficiently resistant and deformable, making it suitable for implantation via minimally invasive surgery, which offers some benefits: *i)* the reduction in incision size, *ii)* the shortening of the operating times, and *iii)* decreased risk of peri‐ and postoperative complications. In addition, it is also less aggressive for the tissues, decreases the recovery time, and, consequently, hospitalization to limit the overall costs. Therefore, to implant the device using minimally invasive surgery, the mesoporous membrane must be initially rolled for insertion and then unrolled into the implantation site thanks to the use of a dedicated trocar. Specific mechanical properties, i.e., elongation at failure of at least 50% and tensile strength of 4 MPa, being close to the surrounding human organs, are required to fit with the minimally invasive procedure.^[^ ^11^ ^]^

Beyond these specifications, the membrane must also be biocompatible and non‐biodegradable, maintaining its integrity and functionality for long‐term use,^[^ ^3^ ^]^ i.e., at least three years in the human body. Thus, the material used to manufacture such a bioartificial pancreas has to meet stringent medical standards, such as USP (United States Pharmacopeia) Class VI and ISO 10 993.^[^ ^12^ ^]^ Furthermore, the membrane must withstand at least one common medical sterilization method, such as moist heat,^[^ ^13^ ^]^ gamma‐irradiation,^[^ ^14^ ^]^ electron beams, or ethylene oxide.^[^ ^15^, ^16^ ^]^ Thermoplastic polyurethane elastomer (PU) appears to be, therefore, a wise choice to meet all these specifications.^[^ ^17^ ^]^

Several techniques have been developed for the manufacturing of mesoporous semi‐permeable membranes, such as track‐etching,^[^ ^18^ ^]^ interfacial polymerization,^[^ ^19^ ^]^ phase inversion methods.^[^ ^20^ ^]^ Recently, polyurethane porous membrane was even manufactured by phase inversion but showing mainly a surface porosity and with the presence of large macropores.^[^ ^21^ ^]^ Thus, although these techniques are well established, they are limited in terms of materials which can be processed with the dedicated porous morphology and also because they often rely on the use of toxic products. Electrospinning process offers the advantage of using a variety of formulations to produce nanofibers, ranging from the classical polymer solutions in organic solvents polymer dispersions such as emulsions or waterborne suspensions. Recently, an environmentally friendly strategy enabling to obtain porous PU membranes from electrospinning by using an aqueous formulation made of a waterborne suspension of PU and a hydrophilic template polymer was developed.^[^ ^22^, ^23^ ^]^ During this green‐electrospinning process, the aqueous formulation is pumped toward a metallic needle being subjected to a high electric potential, resulting in an electric field between the needle and a grounded counter‐electrode, the so‐called collector. When the coulombic forces overcome the surface tension, a liquid jet is emitted from the droplet, taking the shape of the so‐called Taylor cone at the needle tip. During its flight, the jet is subjected to electro‐hydrodynamic instabilities characterized by whipping movements that favor its stretching, the decrease of its diameter, the evaporation of the solvent, and finally, the deposition of a dry and continuous fiber on the collector. The addition of the hydrophilic template polymer in the formulation provides the necessary chain entanglements to prevent jet breakage, ensuring the deposition of a continuous fiber in the form of a non‐woven mat with an open porosity. Playing with the molar mass of the template polymer, its concentration in the processed formulation, its relative amount with the PU nanoparticles, as well as the size of these latter, the rheological properties can be adjusted, allowing control of the morphology of the manufactured fibrous mat. Moreover, by adjusting these parameters, it was possible to fabricate a mat with a PU‐to‐template polymer weight ratio as high as 50.^[^ ^23^ ^]^ These mats show a fibrous structure with an open porosity similar to what it is generally obtained by electrospinning, i.e., pores of diameter in the range of the micrometer.^[^ ^24^ ^]^ Such a pore size is, therefore, two orders of magnitude larger than that expected for a mesoporous membrane.

However, it was observed that a subsequent washing step in water of the PU mat, produced by green‐electrospinning, to remove the hydrophilic template polymer, significantly alters its morphology. Particularly, scanning electron microscopy images showed a drastic decrease of the surface overall pore size.^[^ ^22^ ^]^ Thus, our hypothesis is to take advantage of this morphological remodeling to obtain a PU membrane with pore size in the mesoscale range, i.e., between 2 and 50 nm. In the present article, it is demonstrated that a fibrous mat, obtained via green‐electrospinning of PU, dedicated to long‐term implantation, can be converted into mesoporous membranes with elastomeric mechanical properties through a thermo‐mechanical post‐treatment followed by a washing step. The effects of the post‐treatment parameters and the PU‐to‐template polymer weight ratio on the porous morphology of the membrane (i.e., porosity and pore size characterized by capillary flow porometry) and on the tensile mechanical properties have been investigated. Then, the semi‐permeable properties of the membranes have been characterized by diffusion of FITC‐labelled dextran model molecules as well as FITC‐labelled insulin and IgG antibodies. Finally, in vivo study was conducted on membranes implanted in the extraperitoneal space of rats in order to assess the biocompatibility, i.e., the absence of chronic inflammation and the membrane's integration into the tissue.

## 2 Results and Discussion

### 2.1 Fabrication of a Mesoporous PU Membrane

**Figure 1:**
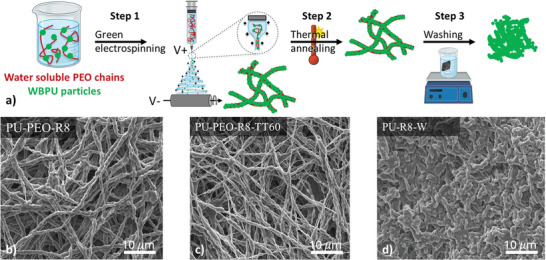
a) Schematic description for the fabrication of a mesoporous PU membrane. SEM picture of b) a mat obtained from PU/PEO = 8 after electrospinning, c) a thermally treated mat obtained from PU/PEO = 8 and an annealing temperature of 60 °C during 120 min, and d) the corresponding PU membrane after washing in water during 48 h.

The mesoporous PU membranes were manufactured in three steps, as shown in **Figure** **1**. During the first step, a fibrous mat is obtained from the green electrospinning of an aqueous PU‐PEOX‐RY formulation, where X stands for the weight concentration of PEO and Y is the PU/PEO weight ratio in the corresponding formulation. It is worth noting that all formulations are very stable during their processing (i.e., at least for more than few days) and that no sedimentation occurs, whatever the PU/PEO ratio as demonstrated by diffusing wave spectroscopy.^[^ ^23^ ^]^ Then, the fibrous mat was subjected to a thermal treatment in order to enhance its cohesion and mechanical strength. Finally, a washing step in water allowed to remove PEO and obtain the final PU mesoporous membrane. The first step was deeply investigated, and several parameters, such as the PU particle size, the PU/PEO ratio, the PEO molar mass, and PEO concentration, were optimized in order to obtain fibrous mats with controlled morphology under stable process conditions.^[^ ^23^ ^]^ Using this green‐electrospinning strategy, mats with PU/PEO weight ratios equal to 4, 6, 8, 12, 16, and 50 were prepared as reported in **Table** **1**.

**Table 1:** Studied formulations and fiber diameters of electrospun mats.

Sample name of electrospun mats	PEO [%w/v]	PU [%w/v]	Solid content [%w/v]	PU/PEO	Fiber diameter [nm]
PU‐PEO4‐R4	4.0	16.3	20.3	4.1	725 ± 460
PU‐PEO4‐R6	4.0	24.0	28.0	6.0	833 ± 448
PU‐PEO4‐R8	4.0	32.0	36.0	8.0	816 ± 345
PU‐PEO4‐R12	4.0	47.8	51.8	11.9	920 ± 340
PU‐PEO4‐R16	4.0	64.0	68.0	16.0	960 ± 208
PU‐PEO2‐R50	2.0	100.0	102.0	50.0	970 ± 720

When a mat having a PU/PEO ratio equal to 8 is further subjected to a thermal treatment and then a washing step, it can be shown that the morphology of the final PU membrane is significantly modified (see Figure 1b–d). As PEO is removed from the washing step, the remaining PU particles coalesce themselves, leading to a compact structure with a significant decrease in porosity as suggested by the SEM pictures. However, if the mat is washed without prior thermal annealing, the PU‐PEO mat is fragmenting showing that this intermediate step is mandatory to maintain the integrity of the final PU membrane.

**Figure 2:**
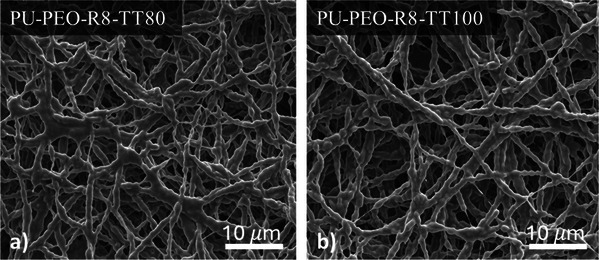
SEM pictures of electrospun mats thermal‐treated at a) 80 °C and b) 100 °C.

The effect of the annealing temperature *T_anneal_* on the fiber structure and mat porosity was investigated by varying it from 60 to 100 °C with a fixed annealing time of 120 min. In the case of *T_anneal_* = 60 °C, SEM images showed that the fiber structure remains unchanged compared to the non‐annealed sample (Figure 1c). Furthermore, the porosity of the mat was also unaffected for such thermal treatment. However, from 80 °C onwards, inter‐fiber fusion begins to appear, as evidenced by the onset of fiber aggregation (**Figure** **2**a). This phenomenon induces a partial coalescence of the fibers resulting from the softening of PEO and PU having respectively a melting temperature of 65.3 and 81 °C (see DSC characterizations, Figure S1, Supporting Information). This morphological reorganization is corroborated by the slight decrease of porosity with increasing annealing temperature. Indeed, a slight decrease of the porosity from values of 69.4 ± 1.6% before treatment to 66.5 ± 0.4% and 66.5 ± 0.8% after thermal annealing at 80 and 100 °C, respectively. This change indicates that the thermal treatment does reduce the inter‐fiber spaces by partially merging the fibers and inducing the shrinkage of the fibrous mat, resulting in slightly increased compactness. However, these changes in the structure of the mat are slight and the fibrous mats exhibit excellent dimensional stability, even during thermal treatment close and above the PEO melting point (60 °C) as also observed in the case of pure PEO fibers.^[^ ^25^ ^]^

**Figure 3:**
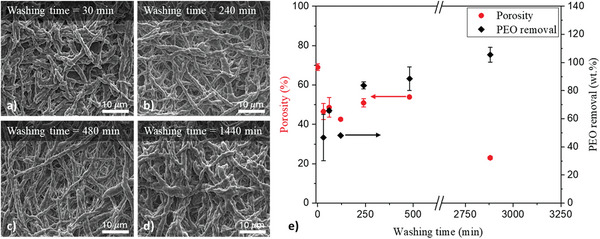
SEM images of PU‐R8‐W membranes obtained from a PU‐PEO‐R8‐TT60 mat subjected to a washing time of a) 30 min, b) 240 min, c) 480 min, and d) 1440 min (24 h). e) Porosity (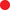) and PEO removal (♦) as function of the washing time.

The effect of washing in water on the morphology of PU‐PEO mats, with PU/PEO = 8 and being previously subjected to thermal annealing at 60 °C during 120 min, has then been thoroughly investigated, particularly through SEM analysis and porosity measurements. This step is crucial because PEO can be easily extracted thanks to its solubilization, a necessary step to achieve the desired properties for biomedical applications. SEM image of **Figure** **3**a reveals a dramatic change in the membrane's morphology after washing a thermally treated mat. Indeed, the fibrous structure collapses into a denser and more compact form. This morphological transformation is attributed to the dissolution and removal of PEO components during the washing process, which was initially responsible for maintaining the fibrous architecture. To confirm the PEO removal, FTIR and DSC were carried before and after washing a PU‐PEO‐R8‐TT60 mat. FTIR did not allow to distinguish specific peaks between PU and PEO. However, DSC analyses reveal the disappearance of the PEO melting peak after a washing step of 48 h, indicating the effectiveness of its removal (see Figure S2, Supporting Information). Thus, as PEO is removed, the remaining PU particles coalesce themselves, leading to a compact structure with a significant decrease in porosity. The impact of the washing time on the morphology and the removal of PEO was investigated by measuring the mass loss as a function of the washing time varying from 30 to 2880 min (48 h). The porosity, which is probably closely tied to the removal of PEO, shows a significant variation over time (Figure 3b). During the first time of washing, the mass of removed PEO increases rapidly, from 46.7 wt.% after 30 min of washing to 83.6 wt.% after 240 min. This partial removal of PEO does visibly alter the surface morphology, as observed in the SEM images (Figures 1 and 3a), and results in a notable decrease in porosity. The progressive decrease in porosity is likely due to the presence of non‐solubilized PEO domains, which gradually reduce the space within the PU fibers. As washing extends beyond 960 min, the PEO is fully removed, with a removal rate of ≈100 wt.%. Moreover, the porosity tends to be stabilized at a value ≈25% from a minimum washing time of 24 h reflecting the transition from a fibrous mat to a porous membrane structure. The thermal post‐treatment and washing steps facilitated thus the production of a stable PU membrane with fine porous characteristics. This study underlines the significance of optimizing the post‐treatment to achieve desired structural properties.

### 2.2 Effect of the PU/PEO Ratio on the Morphology of the Membranes

As the morphology of electrospun mats is significantly influenced by the formulation parameters,^[^ ^23^ ^]^ the effects of varying the PU to PEO weight ratio on the PU membrane morphology obtained after the washing step were studied. PU/PEO ratios varying from 4 to 50 were examined. The *PU‐RY‐W* membranes (where Y denotes the PU/PEO ratio) were prepared from formulations depicted in Table 1. According to the results shown in the previous section, each electrospun mat was subjected to an annealing step at 60 °C for 120 min, followed by a washing step in purified water for 24 h. The porosity of the materials was measured after each step of the fabrication (see **Table** **2**).

**Table 2:** Porosity measurements. *P_ES_* is the porosity of the PU‐PEO‐RY post‐electrospun mats, *P_TT_* is the porosity of the PU‐PEO‐RY‐TT thermal treated mats, and *P_W_* stands for porosity of the final PU‐RY‐W washed membranes.

PU/PEO	*P_ES_* [%]	*P_TT_* [%]	*P_W_* [%]
4	65.8 ± 3.8	64.4 ± 5.7	24 ± 5.7
6	69.8 ± 2.4	71.1 ± 3.7	12.4 ± 5.1
8	70.4 ± 0.2	68.8 ± 0.9	33.4 ± 1.7
12	70.1 ± 0.6	69.4 ± 2.5	19.4 ± 5.9
16	66 ± 1.8	68 ± 1.3	17.9 ± 4.5
50	76.1 ± 0.4	75.6 ± 2.1	9.6 ± 1.2

**Figure 4:**
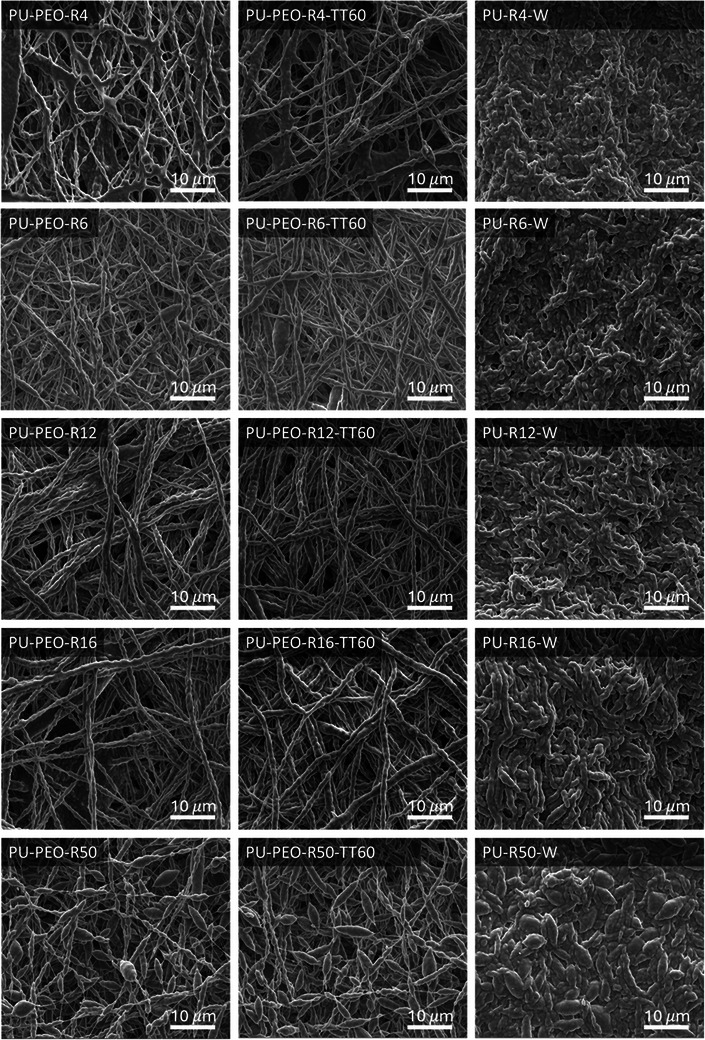
SEM images of the materials obtained from various PU/PEO ratios and after the three steps of fabrication. Post‐electrospun mats (left column PU‐PEO‐RX), annealed samples (middle column PU‐PEO‐RX‐TT60), washed PU membranes (right column PU‐RX‐W).

**Figure** **4** shows the SEM images that illustrate the morphological evolution across the different PU/PEO ratios after each step of fabrication. Post‐electrospinning, the fibers obtained from the lower PU/PEO ratios (4 to 16) appear continuous, well‐defined, and uniform, indicating stable fiber formation due to adequate PEO concentration, which promotes proper chain entanglement. However, at the highest ratio PU/PEO = 50, the fibers display a non‐uniform “bead‐on‐a‐string” structure, which is typically a result of poor chain entanglement caused both by the low PEO content (2 w/v%) and the very high PU content (102 w/v%), which is crucial for stable fiber formation during the electrospinning process. Moreover, Table 1 provides the fiber diameter, which increases according to the PU/PEO ratio. After thermal annealing, the morphology remains largely unchanged across all ratios, showing that the thermal process does not significantly alter the fiber structure. After the washing step, notable changes are observed. For PU/PEO = 50, the SEM images show that the previously observed beads remain agglomerated, forming an even denser and more compact structure than for the lower PU/PEO ratios. The solubilized PEO leaves behind concentrated PU domains, resulting in a more tightly packed and less porous structure compared to the other ratios (Figure 4).

**Figure 5:**
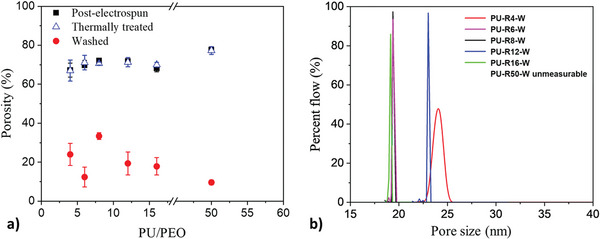
a) Porosity values as function of the PU/PEO ratio for (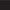) post‐electrospinning, (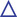) thermal treated, and (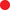) washed membranes. b) Pore size flow distribution of washed PU‐RX‐W membranes at various PU/PEO ratios.

It is observed that the porosity remains relatively constant across all PU/PEO ratios for both the post‐electrospun and thermal‐treated mats, with porosity values of ≈70% (Table 2 and **Figure** **5**a), indicating that the PU content does not significantly alter the overall porosity after all these stages. However, the washing leads to a notable decrease in porosity with increasing the PU/PEO ratio. This decrease is particularly pronounced for PU/PEO = 50, for which the porosity drops to 9.6 ± 1.2% (Table 2 and Figure 5a). This sharp decline in porosity is indicative of a denser structure, which correlates with the SEM observations of agglomerated PU regions after the washing step.

Membranes elaborated from PU/PEO weight ratios varying between 4 and 50 were then characterized by capillary flow porometry. A mean pore size of 2.9 ± 0.4 µm was measured for both post‐electrospun and thermal‐treated mats, whatever the PU/PEO ratio. This value corresponds to what is generally given in the literature.^[^ ^24^ ^]^ However, after the washing step, the pore size decreases drastically to a value of 21 ± 2 nm for PU/PEO ratios varying from 4 to 16 (Figure 5b). This consistency indicated that increasing the PU content in the formulation does not significantly affect the mesoporous structure during the washing process (Figure 4, right column), maintaining the most constricted pore sizes within a narrow and controlled range. However, for PU/PEO = 50 no gas flow was detected during capillary flow porometry measurement, indicating the absence of detectable pore (i.e., either no open pore or pore smaller than the resolution of the porometer of 13 nm). This result corroborates with the very low porosity (9.6 ± 1.2%) as well as the higher fiber density of the mat just after the step of electrospinning (Figure 4) probably explained by the too‐high PU content in regards to PEO.

### 2.3 Toward a Macroencapsulation Device for Type 1 Diabetes Treatment

In order to validate that the washed PU membranes could be used for the elaboration of a macroencapsulation device dedicated to the treatment of type 1 diabetes, the semipermeable properties of the membranes were characterized. More precisely, the membranes were tested for their ability to selectively permit insulin diffusion while rejecting larger IgG antibodies, thereby protecting encapsulated cells from immune attacks. Furthermore, their mechanical properties were assessed to confirm their suitability for rolling and folding, enabling insertion into the patient's body by minimally invasive surgery.

The analysis of the diffusion of fluorescein‐labeled dextran was performed for PU membranes obtained from formulations with varying PU/PEO weight ratios (from 4 to 50). Fluorescein‐labeled dextran molecules of molar mass of 4 and 70 kDa were first used as model molecules of insulin and IgG antibodies, respectively. Indeed, the molar mass of these FITC‐dextran molecules corresponds to diameters of 2.8 and 12 nm, respectively, which is close to the diameter of insulin (2 nm) and IgG antibodies (11 nm).^[^ ^26^ ^]^ Then, the results were corroborated for a given PU‐R8‐W membrane with FITC‐labelled insulin and IgG biomolecules.

**Figure 6:**
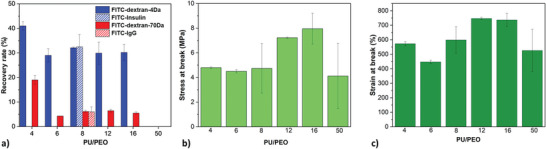
a) Recovery rate of FITC‐dextran of molar mass of 4 kDa (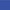) and 70 kDa (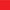) and of FITC‐insulin (only for PU/PEO = 8) and FITC‐IgG (only for PU/PEO = 8) after 24h. b) Stress at failure and c) strain at failure of washed PU‐RY‐W membranes obtained from various PU/PEO ratios. Typical stress‐strain curves can be seen in (Figure S3, Supporting Information).

On one hand, the results reveal that the recovery rate of the 4‐kDa FITC‐dextran is relatively high, with values of ≈30% for PU/PEO varying between 6 to 16 (**Figure** **6**a). It is reminded that the maximum of recovery rate corresponds to a value of 50% (see the Experimental Section). On the other hand, the recovery rate of 70 kDa FITC‐dextran is relatively low, hovering around the targeted threshold of 5%. For PU/PEO = 4, the diffusion of both FITC‐dextran was measured to be higher than the other membranes, at 41 ± 1.7% for the 4 kDa dextran and 19 ± 1.8% for the 70 kDa dextran (Figure 6a). This higher diffusive property could be attributed to instabilities during electrospinning, causing fiber heterogeneity after the electrospinning process, which can remain after washing, where molecules diffusing primarily through areas with defects, even though these heterogeneities are not visible through SEM or porometry analysis. For PU/PEO = 50, no diffusion was observed, whatever the FITC‐dextran molar mass. This result corroborates with the fact that no open porosity was detected by porometry.

In the case of PU/PEO = 8, the diffusion properties of FITC‐labelled insulin and IgG biomolecules were characterized. It is shown (Figure 6a) that the results corroborate with those obtained with FITC‐labelled dextran molecules showing that the mechanism of diffusion is similar and that dextran was a good choice as a model molecule. Regarding IgG, the PU‐R8‐W membranes exhibit diffusion results that are near the threshold of 5% whereas a diffusion of 25% was measured for insulin. These results suggest that the PU‐R8‐W membranes are well‐suited for their intended application in delivering insulin while restricting the diffusion of larger IgG molecules. Considering the membrane's pore size, which is ≈20 nm, and the diameter of insulin (≈2 nm) and IgG molecules (≈12 nm),^[^ ^26^ ^]^ we can better understand the diffusion behavior. The pore size of the membrane is notably closer to the IgG. This size relationship implies that the membranes present a more substantial physical barrier to IgG diffusion compared to insulin, which aligns with the observed diffusion tests.^[^ ^10^ ^]^ As the pores of the membranes are larger than the size insulin, it facilitates the diffusion of these molecules more readily, ensuring thus the therapeutic requirements regarding insulin recovery rate. Additionally, regarding the retention rate, i.e., the percentage of solute (insulin or IgG) that is retained after the diffusion process, the results show no IgG and insulin adsorption on the membranes, suggesting that they maintain semi‐permeable properties all along the diffusion process.

The tensile mechanical properties of the PU membranes obtained from PU/PEO weight ratios between 4 to 50 have been evaluated in regard to the artificial pancreas to be implantable by minimally invasive surgery. The stress‐strain data at failure are presented in Figure 6b. The membranes with ratios of 12 and 16 exhibit higher stress at failure (7.2 ± 0.1 and 7.9 ± 1.2 MPa respectively) as well as higher strain at failure (747 ± 7% and 737 ± 44.7% respectively). These results are correlated with the measured porosity values of these membranes (19.4 ± 5.9% for ratio 12 and 17.9 ± 4.5% for ratio 16) which are lower than those measured for the other PU/PEO ratios, suggesting that lower porosity contributes to enhanced mechanical properties.^[^ ^22^, ^27^ ^]^ The tensile measurements carried out for the membrane obtained from the highest PU/PEO ratio (50) revealed substantial variability but however, showed a decrease of the mechanical properties, despite its lower porosity (9.2 ± 1.2%) than the samples obtained from lower PU/PEO ratios. This result is counterintuitive as lower porosity is typically associated with enhanced mechanical strength. In spite of the relatively high variability in terms of stress and strain at failure, the overall mechanical properties remain within a range that is more than sufficient to withstand the stresses and strains during the fabrication of a macroencapsulation device and the implantation of the membranes in the human body (see the Video S1, Supporting Information showing the handling of a PU‐R12‐W membrane).

**Figure 7:**
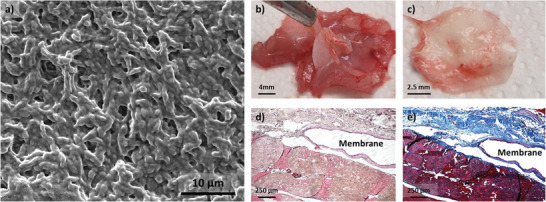
In vivo biocompatibility of PU‐R8‐W membranes. Two membranes were implanted in two rats. a) SEM picture of a PU‐R8‐W after ethylene oxide sterilization before implantation. b) Tissue containing the integrated membrane one month after its implantation. c) Membrane retrieved from the surrounding tissue. The integrity of the membrane is preserved over a month. d) H&E staining of the tissue surrounding the membrane. No cell infiltration was observed. e) Masson's Trichrome staining of the tissue surrounding the disc. Collagen‐positive tissue was observed on the peritoneum side. A thin layer of collagen was observed on the muscle side.

Finally, PU‐R8‐W membranes were implanted in rats in the extraperitoneal space after being subjected to ethylene oxide sterilization. It is worth noting that the integrity and the morphology of the membrane were unaffected by the sterilization step as evidenced by SEM (**Figure** **7**a). The membranes were easily detected after one month of implantation; no degradation was observed (Figure 7b). When the pocket was opened, the membrane was dissected from the surrounding tissue, the totality of the membrane was retrieved (Figure 7c). Interconnections were seen between the membrane and the tissue (Figure 7c). Hematoxylin and eosin (H&E) staining (Figure 7d) and Masson's Trichrome staining (Figure 7e) showed the absence of cell infiltration around the membrane and no fibrotic tissue (only a thin layer of collagen staining on the muscle side was seen). A rearrangement of the tissue on the peritoneum side was observed with collagen fibers and blood vessels. The absence of chronic inflammation (no fibrotic tissue and no cell infiltration) demonstrates the membrane's integration into the tissue, suggesting adequate biocompatibility of the material for further applications.

## 3 Conclusion

In this study, an elastomeric mesoporous polyurethane (PU) membrane suitable for bioartificial pancreas applications, specifically designed for the treatment of type 1 diabetes, was successfully developed. The use of waterborne PU suspension and poly(ethylene oxide) (PEO) in the electrospinning formulation enables the fabrication of a fibrous membrane with an average pore size of 2.9 ± 0.4 µm. Subsequent thermal annealing and washing steps facilitate precise control over the membrane's structural and functional properties, yielding final PU mesoporous membranes with a mean pore size of 21 ± 2 nm. The stress‐strain behavior of the membranes, especially elongation at break, demonstrated adequate elasticity and resistance to failure, ensuring their capability to be rolled and implanted with minimal surgical intervention. These characteristics are particularly significant for reducing the surgical risks and recovery times associated with traditional open surgery.

The diffusion experiments conducted on the fabricated membranes indicated selective permeability, with insulin diffusion reaching a recovery rate of 25%, while immunoglobulin G (IgG) recovery remained below the target threshold of 5%. This selective permeability is crucial for the function of bioartificial pancreas systems, as it allows insulin to diffuse through while blocking larger immune molecules, thus ensuring the protection of encapsulated insulin‐producing cells from immune attacks. The ability of the membranes to retain this functionality under relevant conditions further supports their potential for long‐term biomedical applications.

Additionally, the mechanical tests confirmed that the PU membranes possessed the necessary flexibility and tensile strength required for minimally invasive surgical implantation. The stress‐strain behavior of the membranes demonstrated adequate elasticity and resistance to failure, ensuring their capability to be rolled and implanted with minimal surgical intervention. These characteristics are particularly significant for reducing the surgical risks and recovery times associated with traditional open surgery.

Finally, the membranes maintained their integrity and morphology after ethylene oxide sterilization and one month of implantation in rats, showing no degradation, minimal fibrotic response, and good biocompatibility with tissue integration without chronic inflammation.

In conclusion, the elastomeric PU mesoporous membranes developed in this work show considerable promise for use in bioartificial pancreas devices, offering a robust combination of selective permeability, mechanical stability, and biocompatibility. Future work will focus on long‐term in vivo studies to further validate the performance and durability of these membranes in a clinical setting. Moreover, the adaptation of the manufacturing process and post‐processing steps would allow for the modulation of membrane properties, making them suitable for other advanced biomedical applications, such as tissue engineering or organ‐on‐chip devices.

## 4 Experimental Section

### Materials

Waterborne polyurethane (Baymedix FD103) with a solid content (s.c.) of 59 wt.% and with an average hydrodynamic diameter of the PU particles of 710 ± 40 nm (measured by DLS)^[^ ^23^ ^]^ was provided by Covestro. This PU is dedicated to long‐term implantable biomedical applications thanks to its biocompatibility (*ISO 10 993*, according to supplier datas). Poly(ethylene oxide) (PEO) with molar masses of 900 kDa was purchased from Sigma‐Aldrich. Purified water (milliQ) was employed as solvent for all experiments. Phosphate‐buffered saline (PBS), Fluorescein Iso Thiocyanate (FITC)‐dextran of different molar masses (4 and 70 kDa), FITC‐IgG, bovin serum albumin (BSA) and Tween 20 were purchased from Sigma Aldrich.

### Methods: Preparation of PU‐PEO Formulations

PU‐PEO formulations were prepared for subsequent electrospinning. For each formulation, a stock solution was initially prepared by dissolving PEO powder (m = 0.2 to 0.4 g) in purified water (m = 3.05 to 8.89 g) for 24 h under magnetic stirring at ambient temperature. Subsequently, the waterborne PU suspension (m = 2.71 to 16.95 g) was added into the PEO stock solution and subjected to magnetic stirring for additional 24 h. The weight percentage of PEO relative to the final volume of water was equal to 4%w/v for all formulations except for the weight ratio PU/PEO = 50 for which a PEO ratio of 2%w/v was chosen. The PU/PEO solid weight ratio ranged from 4 to 50. The different formulations are reported in Table 1. In the following part, the formulation samples were named as PU‐PEOX‐RY where X refers to the percentage of PEO in %w/v and Y to the PU/PEO weight ratio. The sample name thus contains all the parameters tuned during the study.

### Methods: Elaboration of the PU Membranes

The PU membranes are fabricated in three steps as shown in Figure 1a. First, a fibrous mat was obtained from the electrospinning of a PU‐PEOX‐RY formulation. Second, the mats were subjected to a thermal treatment, and third, they were washed in water to remove PEO to obtain the final PU membrane once dried. In detail:

*Step 1: Green‐electrospinning*: The first step consists in the fabrication of a fibrous mat from the green electrospinning of a PU‐PEOX‐RY aqueous formulation. A homemade vertical electrospinning setup was composed of a cylindrical aluminum rotating collector having a diameter of 6 cm, three high‐voltage power supplies (Spellman), and two syringe pumps (Harvard). Two needles (18G flat‐tip) were placed diametrically in relation to the collector. The ambient air conditions were controlled and fixed to 21 ± 1 °C and 25 ± 5% of relative humidity. The flow rate was fixed at 1.5 mL h^−1^ per needle, and the needle‐to‐collector distance at 18 cm. The rotation speed of the collector was fixed at 100 rpm. The voltage applied at the collector and the emitters were set respectively to −4 and 20 kV, and the duration of electrospinning was 180 min. The collector was covered with silicone‐coated paper to facilitate the peeling of the fibrous mat. The resulting mats are named PU‐PEO‐RY, with Y being the PU/PEO weight ratio.

*Step 2: Thermal treatment of the electrospun fibrous mats*: The mats were exposed to a thermal treatment in an oven at a temperature of 60, 80, or 100 °C during 120 min. These parameters were determined after studying their effect on the morphology of the mat. The resulting mats are named PU‐PEO‐RY‐TTZ, where Y is de PU/PEO ratio and Z is the annealing temperature.

*Step 3: Washing step*: With the exception of the study of the effect of washing time on the morphology and porosity of final membranes, PU‐PEO‐RY‐TT60 mats were immersed in a tank filled of purified water (milliQ) at room temperature for 24 h, with orbital stirring provided by a 3D rotary shaker at 10 rpm (Polymax 1040, Heidolph Instruments). To ensure the complete removal of residual PEO, the water was replaced twice during all the process. Subsequently, the PU membranes were removed from the solvent tank and naturally dried in air for 24 h prior to characterization. The resulting membranes were named PU‐RY‐W.

After drying, the removed mass of PEO was measured by comparing the initial ($m_{i}$) and final ($m_{f}$) masses of the samples, and quantified using the following equation:

(1) $$\%PEO_{removed}=\left(\frac{m_{i}-m_{f}}{m_{i}\times \left(\frac{\%PEO_{solid}}{100}\right)}\right)$$

where $\%PEO_{solid}$​ is the percentage of solid PEO in the electrospun mat. This method relies on a key assumption: it presumes that the difference in weight before and after washing is only due to the PEO removal, with no PU polymers being lost during the washing process.

### Methods: Scanning Electron Microscopy Characterization (SEM)

Samples were cut into pieces of 4 × 4 mm^2^ and placed on a conductive carbon tape on the microscopy stub. Electrospun samples were sputter coated with a thin gold layer prior to observation (Q150RS, Quorum technologies) for 2 min. Scanning electron microscopy (SEM, TESCAN Vega 3 LMU) was used in high vacuum mode using an accelerating voltage of 5 kV and a working distance of 7 mm. The average diameter of the nanofibers was calculated by measuring 100 diameters nanofibers distributed on two different SEM images with the ImageJ analysis software.

### Methods:Porosity Measurements

The porosity P was estimated for each sample after each step of the process (i.e., after electrospinning, thermal annealing and washing). Three samples were prepared and subjected to all process steps. First, circular samples with a diameter of 10 mm were precisely cut and weighted to get their surface S and mass m, respectively. The thickness $e_{tot}$ of the samples was systematically measured by using a digital indicator with a 5 mm flat measuring contact point (Absolute Digimatic ID‐C; Mitutoyo; accuracy 0.001/0.01 mm; F < 2.5 N).

After the two first steps (i.e., electrospinning and the thermal steps), fibers contain both PU and PEO polymers, which must be considered in the calculation of the porosity. Furthermore, it is assumed that the experimental weight ratio between the PU and PEO in the fibrous mat is equal to $\frac{m_{PU}}{m_{PEO}}=Y$, i.e., the PU/PEO weight ratio of the corresponding processed formulation. Thus, the total mass of the sample is $m\;=\left(1+Y\right)\times m_{PEO}$. The densities of the polymers are respectively equal to $\rho_{PU}$= 1.05 g cm^−3^ and $\rho_{PEO}$= 1.21 g cm^−3^. The porosity P of the mat then can be calculated from the following equation:

(2) $$\begin{array}{ccc} P_{before\;washing} & = & \frac{V_{pores}}{V_{tot}}=1-\;\frac{V_{material}}{V_{tot}}=1-\frac{1}{Se_{tot}}\;\left(\frac{m_{PU}}{\rho_{PU}}+\frac{m_{PEO}}{\rho_{PEO}}\right)\\ & = & 1-\frac{m/\rho_{PU}}{Se_{tot}}\left(\frac{Y\;m_{PEO}}{m}+\frac{\rho_{PU}}{\rho_{PEO}}\frac{m_{PEO}}{m}\right) \end{array}$$

Thus,

(3) $$P_{before\;washing}=1-\frac{m/\rho_{PU}}{Se_{tot}}K_{\rho }$$

With $K_{\rho }$, the following parameter:

(4) $$K_{\rho }=\left(Y+\frac{\rho_{PU}}{\rho_{PEO}}\right)\;\frac{1}{1+Y}$$

The parameter $K_{\rho }$ is a correction factor considering the relative composition of the fibers.

The porosity $P_{membrane}$ of the mesoporous single‐component PU membranes, obtained after the washing step, can be easily calculated with Equation (5).

(5) $$P_{membrane}=1-\frac{m_{PU}/\rho_{PU}}{S_{PU}e_{tot}}$$

### Methods: Capillary Flow Porometry CFP

A POROLUX Revo capillary flow porometer (Porometer) was used to measure the through pore size and pore size distribution. It is based on the gas‐liquid displacement. The wetting liquid used was the Porefil, a perfluorether liquid with a surface tension of 16 mN m^−1,^ and the gas was nitrogen. The pore size distribution was obtained from the Young‐Laplace equation:^[^ ^28^ ^]^

(6) $$D=\frac{4\gamma \cos\left(\theta \right)}{\Delta P}$$

where D is the pore diameter, γ and θ the surface tension and contact angle of the wetting liquid, respectively. ΔP is the differential pressure. The use of Equation 5 assumes that the pores have an ideally circular cross‐section. In this technique, the liquid to impregnate the pore of the membrane is fully wetting, which implies that cos(θ) is equal to 1.

Three circular disks of 25 mm were cut for each sample to ensure uniformity across all tests. The pore size distribution can be calculated from the ratio of the respective volume gas flow rates through the dry and the wet media obtained at the same ΔP. In the wet measurement phase, the sample was saturated with a wetting liquid, and nitrogen was used. CFP is a liquid extrusion technique. The method used is a pressure step method, i.e., the gas pressure gradually increases, and 60 steps maintained 20 s were applied. It allows for more accurate data collection on the pore sizes by taking into account the tortuosity discrepancies of pores of same diameter. It is noteworthy that the method enables to obtain the most constricted part of the pore size. Once the wet measurement is complete, the dry measurement follows. Here, only nitrogen gas is passed through the dry sample, and the data are recorded at 25% of the wet measurement points. The pressure applied during these measurements is controlled within an accuracy of 1%. Additionally, measurements are taken as soon as the pressure value is stabilized for 2 s.

### Methods: Uniaxial Tensile Strength

The mechanical properties of the washed PU‐RX‐W membranes were evaluated using an MTS 1/M testing bench (MTS systems) with pneumatic jaws adapted to the thickness of the membranes and equipped with a 100 ± 1 N load cell. Displacement speed 9 mm.min^−1^ (corresponding to a strain rate of 0.006 s^−1^) was used for all the tests. Each test specimen was obtained into a “dog‐bone” shape by laser cutting. The total length of each sample was 60 mm, the ends of the samples held in the jaws of the traction machine had a length of 15 mm and a width of 15 mm. The thickness of the samples was measured by using a digital indicator with a 5 mm flat measuring contact point (Absolute Digimatic ID‐C; Mitutoyo; accuracy 0.001/0.01 mm; F < 2.5 N). The thickness of the PU‐RX‐W membranes was 134 ± 8 µm. The usable size being stretched during the test had an initial length of 25 mm and a width of 10 mm. Tests were performed on three samples for all conditions.

### Methods: Diffusion Tests

For tests involving FITC‐dextran and FITC‐IgG, a buffer solution composed of PBS with 0.05% Tween 20 and 0.1% BSA was used. For FITC‐insulin, a buffer solution consisting of distilled water and 0.9% NaCl was used. The FITC‐IgG and FITC‐dextran solutions were prepared at concentrations of 13 µg mL^−1^ in their respective buffer solutions, while FITC‐insulin (Umulin) was used at a concentration of 10 IU mL^−1^. All preparations were made by avoiding light exposure and stored at 4 °C. The retention rate for FITC‐dextran, FITC‐IgG, and FITC‐insulin was determined according to the protocol described by Magisson et al.^[^ ^10^ ^]^ The tests were conducted in vitro using a homemade vertical diffusion chamber with two compartments separated by the membrane to be characterized. The molecules of interest (i.e., FITC‐dextran, FITC‐IgG, or FITC‐insulin) were solubilized in a buffer solution, and 3 mL were put in the upper compartment (donor). The lower compartment (receiver) was filled with 3 mL of pristine buffer solution. The diffusion chambers were then incubated for 24 h at 37 °C in an oven. Each test was triplicate. Only one molecule is tested at a time. At the end of the test, samples are taken from both compartments to determine the concentration of molecules of interest thanks to the established calibration curves and thus calculate the recovery rate of the molecule through membrane by fluorescence spectroscopy. Since the volumes of the upper and lower compartments are identical, recovery rate (expressed as a percentage) was calculated as the ratio of the molecule concentration in the lower compartment to the sum of the concentrations measured in both the upper and lower compartments. Maximum of recovery rate was achieved when equal quantities of the molecule were detected in the upper and lower compartments, corresponding to a value of 50%. A molecule was considered to be rejected by the membrane if the recovery rate was below 5%. Three different samples were used for each condition. This protocol was routinely used for quality control of membrane batches and has successfully undergone method validation feasibility in accordance with the World Health Organization guidelines on good manufacturing practices (Section 15).

### Methods: In vivo Assays

In vivo protocols were approved by the local ethics committee (CREMEAS, Strasbourg, France, No. 2 019 030 412 045 725 and French Ministry of Research). Male Wistar rats (Strain: Crl:WI(Han), Charles River labs, L'Abresle, France) weighing 125–150 g corresponding to an age of 5 weeks were used.

The membranes were cut into 1cm^2^ discs. Two discs were implanted in two rats in the extraperitoneal cavity. Implantation was performed under isoflurane anesthesia. The rats were placed in dorsal decubital position, laparotomy, skin, and muscle incision along to the white line were done. Two extraperitoneal pockets were formed by the separation of the muscle from the peritoneal membrane. The disc was inserted, and then the muscle and cutaneous plan were sutured. One month post‐implantation, the rat was sacrificed by pentobarbital 150 mg kg^−1^ injection and tissues surrounding the membrane were retrieved.

For the first rat, photographs of the surrounding tissue and of the membrane were taken. The second rat tissues were fixed with 4% paraformaldehyde solution and embedded in paraffin for sectioning. The sections were dewaxed, and after staining for hematoxylin and eosin (H&E) staining with hematoxylin solution for 3–5 min, the sections were washed with 1% acidic ethanol (1 mL of hydrochloric acid, 99 mL of 70% ethanol), rinsed with running water, stained with eosin solution for 0.5–1 min, dehydrated, mounted, and placed under a light microscope for observation and imaging. The tissue was also stained with Masson's trichrome. The sections were dewaxed and placed in potassium dichromate overnight, followed by hematoxylin, Ponceau red acid fuchsin, phosphomolybdic acid, and aniline blue staining, dehydration, mounting, and imaging under a light microscope.

## Conflict of Interest

The authors declare no conflict of interest.

## Supporting information



Supporting Information

Supplemental Video 1

## Data Availability

The data that support the findings of this study are available from the corresponding author upon reasonable request.
