# Supplementary material for: Mesoporous Semi‐Permeable Flexible Polyurethane Membranes: Advancing Bioartificial Pancreas Design for Type 1 Diabetes Treatment
Source: Macromol Rapid Commun. 2025 Feb 14;46(13):2500049. doi: 10.1002/marc.202500049 (PMC12227223; doi:10.1002/marc.202500049)
Supplement: Supplementary file 1 — Supporting Information [file MARC-46-2500049-s001.pdf]

Macromolecular Rapid Communications

Supporting Information

for *Macromol. Rapid Commun.*, DOI 10.1002/marc.202500049

Mesoporous Semi-Permeable Flexible Polyurethane Membranes: Advancing Bioartificial Pancreas Design for Type 1 Diabetes Treatment

*Bryan Gross, Emeline Lobry, Séverine Sigrist, Elisa Maillard, Jordan Magisson, Charles-Thibault Burcez, Manuel Pires, Anne Hébraud and Guy Schlatter**

Supporting Information

Mesoporous Semi-Permeable Flexible Polyurethane Membranes: Advancing Bioartificial Pancreas Design for Type 1 Diabetes Treatment

Bryan Gross,[1] Emeline Lobry,[1] Séverine Sigrist,[2] Elisa Maillard,[2,3] Jordan Magisson,[2] Charles-Thibault Burcez,[2] Manuel Pires,[2] Anne Hébraud[1] and Guy Schlatter[1,]*

[1] Institut de Chimie et Procédés pour l'Energie, l'Environnement et la Santé, ICPEES UMR 7515, CNRS, Université de Strasbourg, Ecole Européenne de Chimie, Polymères et Matériaux, 25 rue Becquerel, 67087 Strasbourg Cedex 2, France.

[2] Defymed, 9 rue Albert Calmette, 67200 Strasbourg, France

[3] Institut National de la Santé et de la Recherche Médicale, UMR_S 1121, Strasbourg, France, Université de Strasbourg, Faculté de Chirurgie Dentaire, Strasbourg, France

Email of the corresponding author: guy.schlatter@unistra.fr

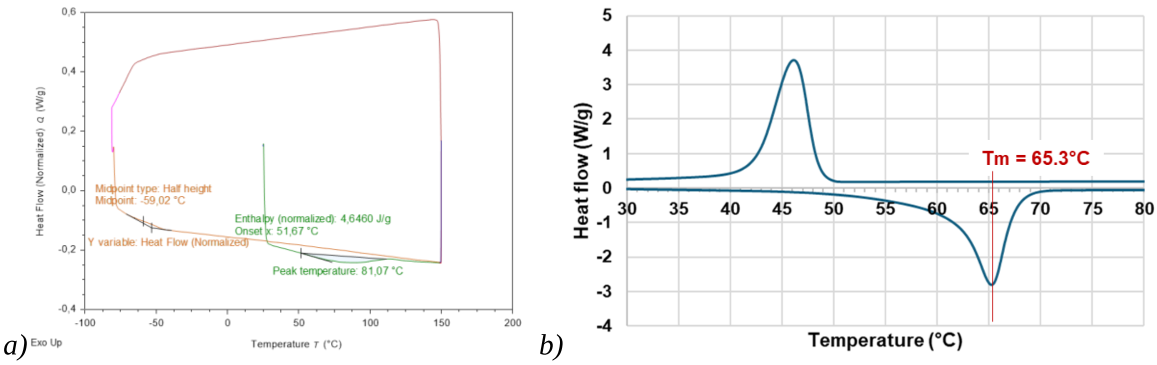

Figure S1. *a) DSC of the studied PU. b) DSC of the studied PEO (molar mass of 900 kg/mol).*

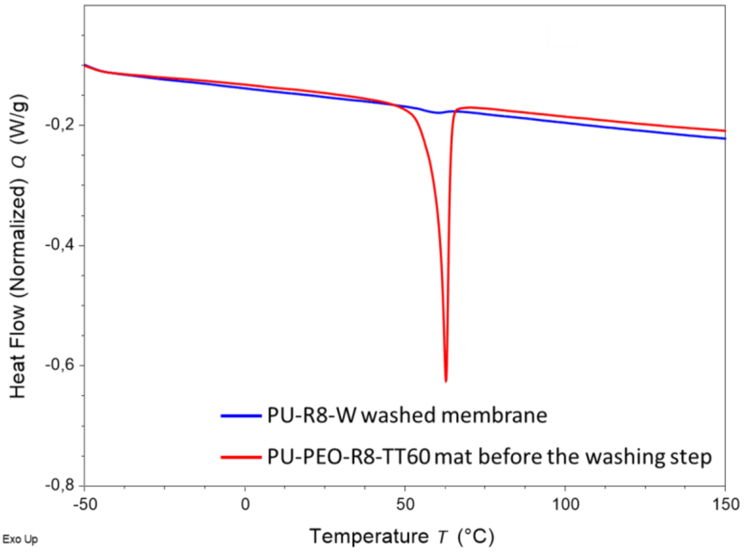

Figure S2. *DSC of a PU-PEO-R8-TT60 mat (red) and the PU-R8-W membrane obtained after the washing step (blue).*

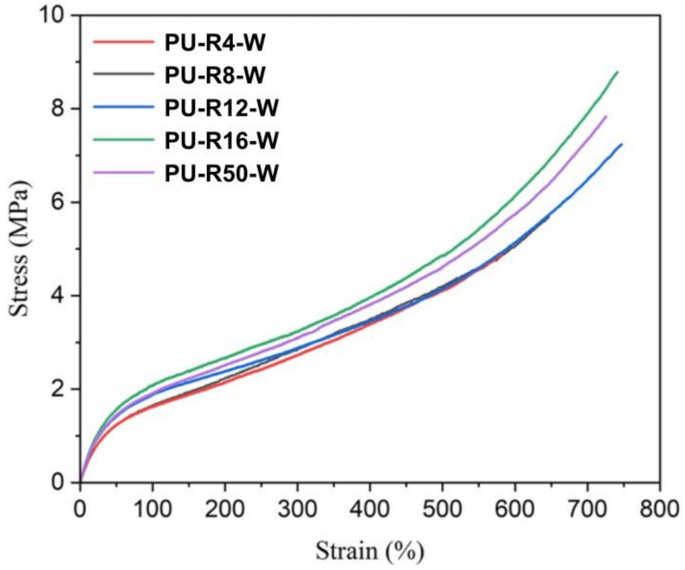

Figure S3. *Typical stress-strain curves of PU membranes.*
